# 2033. Use of Sterile, Single-Use Bronchoscopes Reduces Procedure-Associated Readmissions Rates and Eliminates Potential Device Cross-Contamination

**DOI:** 10.1093/ofid/ofac492.1656

**Published:** 2022-12-15

**Authors:** John H Garrett

**Affiliations:** University of Louisville School of Medicine Division of Infectious Diseases, Atlanta, Georgia

## Abstract

**Background:**

Improper reprocessing of reusable bronchoscopes poses a serious patient safety risk. The risk of patient infection resulting from use of contaminated bronchoscopes has been reported to be 2.8%. By virtue of being sterile, SFB eliminates device-related infection risk, subsequent readmission rates, and associated costs. Readmissions following bronchoscopy may occur for many reasons, but most frequently are due to infection, bleeding, or pain. These infections are preventable and can be eliminated with the use of a sterile, single-use bronchoscope when the device is used according to the manufacturer’s instructions for use.

**Methods:**

Bronchoscopies with SFB and RFB were identified along with their corresponding readmission information in the Premier Healthcare Database from 2016- 2019. A logistic regression analysis was conducted with 30-day readmission as the dependent variable. Independent variables included use of a RFB, gender, race, age, payer, and discharge status. For the inpatient setting, the 3M™ APR DRG Severity of Illness classification was included as an independent variable.

30 Day Readmissions

**Figure:**
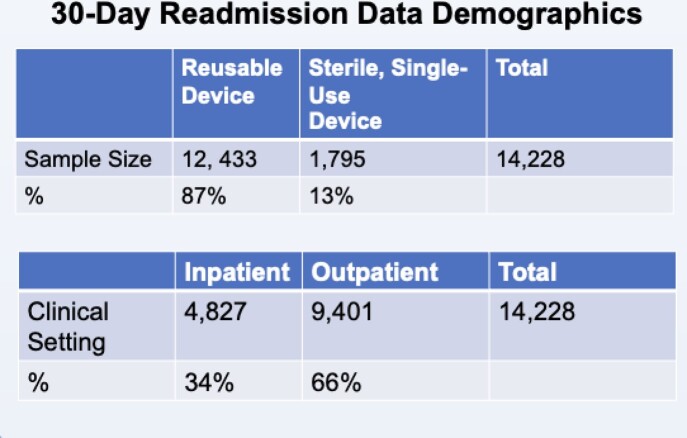

**Results:**

A total of 14,228 procedures were identified, of which 1,795 used SFB and 12,433 used a RFB. In the inpatient setting, the RFB group was ∼2.5 times more likely to be readmitted within 30 days compared to the SFB group (OR=2.5, p< 0.01), controlling for patient demographics and risk. In the outpatient setting, the RFB group was ∼1.5 times more likely to be readmitted than the SFB group (OR=1.5, p >0.05). Across all settings, the RFB group was ∼2.3 times more likely to be readmitted than the SFB group (OR=2.3, p< 0.01). The inability to fully risk-adjust in the outpatient setting may have been a factor in the smaller OR and lack of statistical significance; this limitation will be addressed with further analysis that includes an outpatient severity of illness classification variable.

**Figure:**
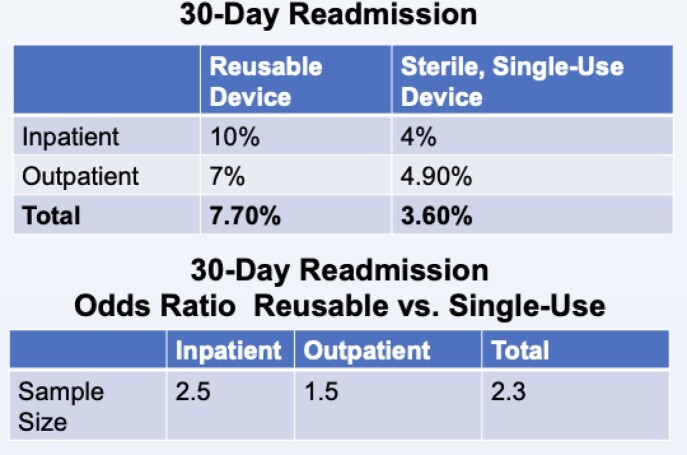

**Conclusion:**

Patients undergoing bronchoscopy typically have significant comorbidities which increase their risk for the development of potential device-related infections. To reduce these risks, the use of a sterile SFB should be considered to eliminate reprocessing failures, improve overall operational efficiency, and reduce potential acquisition of healthcare-associated infections.

**Disclosures:**

**John H. Garrett, PhD, MSN, MPH, MBA, FNP-BC, FNAP, FSHEA, FIDSA**, Aerobiotix: Advisor/Consultant|Ambu: Advisor/Consultant|American Academy of Nurse Practitioners: Honoraria|Clorox Pro: Advisor/Consultant.

